# 679 Utilization of a Palliative Care Assessment Tool Within a Verified Adult Burn Center

**DOI:** 10.1093/jbcr/iraf019.308

**Published:** 2025-04-01

**Authors:** Caitlin Sayers, Jennifer Embree, Shawna Thomas, Deidra Bedgood, Brett Hartman, Leigh Spera, Claire Willard, Rafael Rosario, Cortni Grooms

**Affiliations:** Richard M. Fairbanks Burn Center; Eskenazi Health and Indiana University School of Nursing; Eskenazi Health; Eskenazi Health; Eskenazi Health; Indiana University; Eskenazi Health; Eskenazi Health; Eskenazi Health

## Abstract

**Introduction:**

Palliative care is a commonly used consultation service in the trauma world, however, interestingly, only 2% of burn patients, a subset of trauma patients, have the luxury of a palliative care consult during hospitalization. The early introduction of palliative care during a burn survivor’s hospitalization may reduce miscommunication, the feeling of hopelessness, and incongruent care coordination. The purpose of this study is to evaluate the incidence of palliative care consultations on a verified adult burn center by comparing pre- and post- intervention data following the implementation of a palliative care assessment tool, the Palliative Care Problem Severity Score (PCPSS).

**Methods:**

A retrospective chart review was conducted to analyze the pre- and post-intervention groups. Specifically, 57 patient charts were reviewed for the presence of palliative care in the three months leading up to project implementation. After the project was initiated and the PCPSS was implemented, post-intervention chart reviews were completed on a total of 33 patients. A Fisher’s Exact Test was used for data analysis. Demographic data was also collected for the pre- and post-intervention groups for further comparison.

**Results:**

A Fisher’s Exact Test was conducted to appropriately measure pre- and post-intervention data. The test was able to sufficiently evaluate the effectiveness of the PCPSS, rendering statistically significant results (p = 0.029). Objectively, only one out of 57 patients (1.75%) received a palliative care consultation in the pre-intervention group while five out of 33 patients (15.15%) received a palliative care consultation in the post-intervention group, resulting in a 13.4% increase in palliative care consultations after the implementation. The PCPSS was able to reduce the time from patient admission to initial palliative care consultation from 44 to 2.8 days.

**Conclusions:**

Advanced Practice Providers can be at the forefront of the palliative care movement within the burn specialty by streamlining the use of the PCPSS within their practice. The results of the chosen palliative care assessment tool surpassed the expectations of the initially proposed project, and its effects will linger in the lives of the burn patients whose outcomes were optimized by the introduction of palliative care early in the acute phase of their burn injury.

**Applicability of Research to Practice:**

Ultimately, the implementation of the PCPSS is the first step towards formalizing a palliative care consultation process within adult verified burn centers. The project’s results can formalize a palliative care consultation standard within a verified adult burn center while bridging the identified literature gap between two specialty services that, if united, will provide limitless resources to burn survivors from the time of injury to rehabilitation.

**Funding for the Study:**

N/A

